# Age of onset of nicotine use and severity of nicotine addiction symptoms are associated with hippocampal volume in late adolescents and emerging adults

**DOI:** 10.3389/fradm.2025.1532450

**Published:** 2025-06-10

**Authors:** Joseph P. Happer, Kelly E. Courtney, Rachel E. Baca, Gianna Andrade, Qian Shen, Thomas T. Liu, Joanna Jacobus

**Affiliations:** 1 Department of Psychiatry, University of California San Diego, San Diego, CA, United States; 2 Department of Neurosciences, University of California San Diego, San Diego, CA, United States; 3 Center for Functional MRI, University of California San Diego, San Diego, CA, United States; 4 Department of Radiology, University of California San Diego, San Diego, CA, United States

**Keywords:** hippocampus, age of onset, nicotine, vaping, adolescent, emerging adult

## Abstract

**Background:**

Despite declining use of traditional combustible cigarettes, the use of nicotine and tobacco-related products (NTPs) remains high among adolescents and emerging adults largely due to the use of e-cigarettes. Adolescents and emerging adults who initiate e-cigarette use reach comparable indices of nicotine dependence as traditional cigarette smokers and can report symptoms of dependence even before developing a pattern of daily use. Symptoms such as craving, positive and negative reinforcement, and biological markers of toxicity are closely linked to the development and persistence of substance use problems. Adolescents/emerging adults may transition to dependence more quickly than adults, and the age of onset of regular NTP use is a highly predictive risk factor for future use and problems. Within the brain, the hippocampus is particularly sensitive to the effects of nicotine and may play a role in the transition from NTP initiation to more habitual and even problematic use.

**Methods:**

A cross-sectional sample of healthy, NTP-using late adolescents/emerging adults (*N* = 86) ages 16–22 completed a structural MRI to examine whether subjective nicotine craving, stronger positive and negative reinforcement, elevated cotinine levels, and earlier age of onset of regular nicotine use would be associated with hippocampal volumes.

**Results:**

Across measures of nicotine addiction, linear regression models revealed an interaction between symptoms and age of onset of regular use. A general pattern emerged such that greater symptom severity and younger age of onset of regular use was associated with larger hippocampal volumes.

**Conclusions:**

These findings provide potential insight into the relationship between late adolescent/emerging adult brain health and a risk factor for NTP initiation and symptoms of nicotine addiction. Greater understanding of these interactions is essential for informing prevention, intervention, and public health policy.

## Introduction

While the use of combustible cigarettes has declined among late adolescents and emerging adults [AEAs, (1, 2)], the prevalence of nicotine and tobacco-related products (NTPs) remains high (1, 3, 4), largely due to the increased use of e-cigarettes (2, 5). Although e-cigarettes were initially marketed as tools for cigarette cessation (6, 7), AEAs have frequently been targeted with digital advertising by tobacco companies (8, 9), which may contribute to reductions in perceived risk and more favorable attitudes towards e-cigarette use (10–12). E-cigarettes provide similar or possibly greater nicotine delivery per puff as compared to combustible cigarettes (13), and vaping further allows for easy consumption across the day leading to increased use, intensity, and nicotine exposure (14, 15). AEAs who initiate use of e-cigarettes reach comparable indices of nicotine dependence as cigarette users (16–18) highlighting the highly addictive nature of nicotine for AEAs (19–21). Notably, use of e-cigarettes among AEAs has been associated with problematic substance use including alcohol and cannabis misuse (22–25). Therefore, understanding the associations between known risk factors for the initiation of e-cigarettes and other nicotine use, the progression to nicotine dependence, and their impact for brain health is crucial for informing prevention, intervention, and public health policy.

Craving, positive and negative reinforcement (e.g., pleasurable effects, escaping unpleasant states), and biological markers of toxicity (i.e., the accumulation of harmful metabolites in the body) are closely linked to the development and persistence of substance use problems. Substance dependence can generally be defined as intense cravings for the substance of choice, development of tolerance, and loss of autonomy over use despite potential negative consequences (26). Consistent with this definition, reports of initial symptoms of nicotine dependence among AEAs have included intense craving or desire to use, feelings of loss of control, withdrawal, and tolerance (19, 27–29). These symptoms have been associated with increased risk for continued and even escalated NTP use (19, 27–30). Initial pleasurable experiences with NTPs can similarly predict future use as well as severity of dependence symptoms among AEAs (28). This pattern maps on to models of addiction in which substances such as nicotine are often initiated for their hedonic effects and continued due to positive reinforcement of those experiences (26). Repeated use of nicotine can then lead to tolerance and the need for greater consumption (26). Consistent with more intense NTP use, higher levels of cotinine, the primary metabolite of nicotine, have been associated with greater symptoms of dependence in AEAs, potentially reflecting greater physiological dependence and thus neurobiological changes (31–33). This is particularly concerning as AEAs may report dependence symptoms even before developing a pattern of daily use (20, 34), suggesting heightened sensitivity to the effects of nicotine (35).

Age of onset of regular substance use is also a highly predictive risk factor for future use and dependence (36–40). Individuals who regularly engage in NTP use at younger ages are at increased risk of developing nicotine dependence (36, 39, 40) and may transition to dependence more quickly than adults and even older AEAs (37, 39). Indeed, AEAs may develop nicotine dependence even after minimal exposure (19–21, 27, 28, 34), and nicotine exposure during adolescence/emerging adulthood may uniquely impact brain health compared to older adults (35, 41–43), underscoring the heightened sensitivity of this developmental period to substance use (44). This may be related to nicotine’s binding to nicotinic acetylcholine receptors (nAChRs), which can alter nACHRs expression and function (42). These receptors are distributed throughout the brain (42) and may play a role in the gray and white matter morphometric changes observed in association with AEA NTP use (45–51). In particular, the hippocampus is dense with nAChRs (42) and is involved with reinforcement learning and episodic memory of rewarding stimuli (52), which may be particularly heightened in AEAs (41). Nicotine may also enhance dopaminergic transmission within the nucleus accumbens and dorsal striatum, regions heavily implicated in reward processing and the development of substance dependence (41, 52).

We recently reported greater cumulative 6-month NTP use was associated with larger bilateral hippocampal volumes in a sample of AEAs (49). Given greater cumulative use is associated with more severe dependence (19, 27–30) as well as younger age of onset of use (36, 37, 39, 40), in this report we sought to examine these relationships with hippocampal volumes within the sample of AEAs who had initiated regular NTP use. More specifically, we hypothesized that indicators of problematic NTP use, including greater subjective nicotine craving, stronger positive and negative reinforcement, elevated cotinine levels, and earlier age of onset of regular NTP use would be associated with larger hippocampal volumes.

## Methods

### Participants and procedures

Eighty-six participants were selected for this analysis from a larger study on the effects of nicotine and cannabis co-use on brain structure and function during late adolescence/emerging adulthood. As previously reported (48, 53), participants were recruited via flyers posted physically and electronically at schools, community colleges, four-year universities, and social media sites targeting San Diego County. Recruitment was stratified based on use of NTPs, cannabis products, or both during the previous 6-month period to ensure variability in NTP and cannabis use.

Exclusion criteria included >10 lifetime episodes of illicit substance use; lifetime DSM-5 psychiatric diagnoses other than tobacco and/or cannabis use disorder; acute influence of cannabis or alcohol use at study visit; use of any psychoactive medications; major medical problems; MRI contraindications; or history of prenatal substance exposure or developmental disability.

Participants completed a single 4-hour session consisting of a battery of interviews, self-report assessments covering demographic information, mental health, substance use, and neurocognitive functioning, which was followed by an MRI session. Before beginning the study session, all participants gave written informed consent (≥18 years old) or parental consent and participant assent (<18 years old). Participants were asked to refrain from using cannabis and alcohol 12 h prior to the appointment, which was confirmed with oral fluid, urine, and breathalyzer. Urine samples were used to confirm abstinence from illicit substances. Participants abstained from caffeine for at least 30 minutes prior to MRI scanning. They were not required to abstain from NTP use to avoid nicotine withdrawal effects during testing. Time of last NTP use was documented. All procedures were approved by the University of California, San Diego Human Research Protections Program.

### Measures

Demographic data (e.g., age, sex at birth, race/ethnicity, education) were obtained from a psychosocial interview. To assess quantity and frequency of NTP and cannabis use, the Customary Drinking and Drug Use Record structured interview (54) was used, including a modification to include additional nicotine and cannabis questions (55–57). Lifetime use of nicotine, cannabis and alcohol were estimated in terms of independent episodes, allowing for multiple uses to be reported within a single day (e.g., first thing in the morning, again before bed). Participants were asked to provide additional details related to each substance reported including age at first use and onset of regular (at least weekly) use.

As part of the assessment, participants completed a range of self-report questionnaires related to their NTP use experiences. Severity of nicotine dependence was assessed using the Hooked on Nicotine Checklist [HONC, (58)]. They completed an adapted Smoking Consequences Questionnaire [SCQ, (59)] with questions specific to e-cigarette use. Four subscales can be calculated from the SCQ: negative consequences, positive reinforcement, negative reinforcement, and weight control. For the purposes of this study, only the positive and negative reinforcement subscales were included in analyses. To examine nicotine craving, participants completed the 10-item version of the Questionnaire on Smoking Urges, which was modified to reflect both cigarette and vaping urges, and a total score was computed [QSU, (60, 61)]. Acute nicotine exposure was examined through quantification of urine cotinine levels, which is nicotine’s major metabolite (quantification conducted by Redwood Toxicology). Cotinine values were capped at 500 ng/ml per Redwood Toxicology’s standard procedures. See Table 1 for a complete description of the sample demographics and substance use characteristics.

### Imaging acquisition and processing

Participants were scanned on a 3.0 Tesla GE Discovery MR750 scanner with a 32-channel receive head coil at the UCSD Center for Functional MRI. A high-resolution T1-weighted anatomical fast spoiled gradient echo (FSPGR) scan was acquired with TI/TE/TR = 1,060/2/2,500 ms, 256 × 256 matrix, flip angle = 8°, FOV = 256 mm, 1.0 mm^3^ voxels. Brain images for each participant were spatially normalized, field-bias corrected, and segmented using the Freesurfer pipeline [version 6.0, (62, 63)]. To identify errors made during the Freesurfer reconstruction process, one rater (QS), blind to participant characteristics, followed the reconstruction procedures to correct any errors made during the cortical and subcortical reconstruction process. This involved verification of the automated skull stripping and a slice-by-slice inspection of the gray/white and gray/cerebral spinal fluid surfaces. Modifications to the surfaces were made as necessary to correct for tissue misclassifications (e.g., residual dura mater classified as cortex). Right and left hippocampal volumes and an estimate of total brain volume (“BrainSegVolNotVent”) were extracted for analyses.

### Data analyses

Data analyses were conducted using R (v4.3.2). Estimates of bilateral hippocampal volumes were examined using individual linear regressions that modeled the interaction between age of onset of regular NTP use and four indices of nicotine addiction severity including: (1) severity of acute nicotine exposure quantified in urine cotinine values; (2) the self-reported positive and negative reinforcing effects of nicotine (SCQ subscales); (3) the craving and urge to use NTPs (QSU total); and (4) nicotine dependence symptoms (HONC total). Total brain volume, current age, sex assigned at birth, and lifetime alcohol, cannabis, and NTP use episodes were included in the models as covariates.

## Results

### Cotinine

**FIGURE 1:**
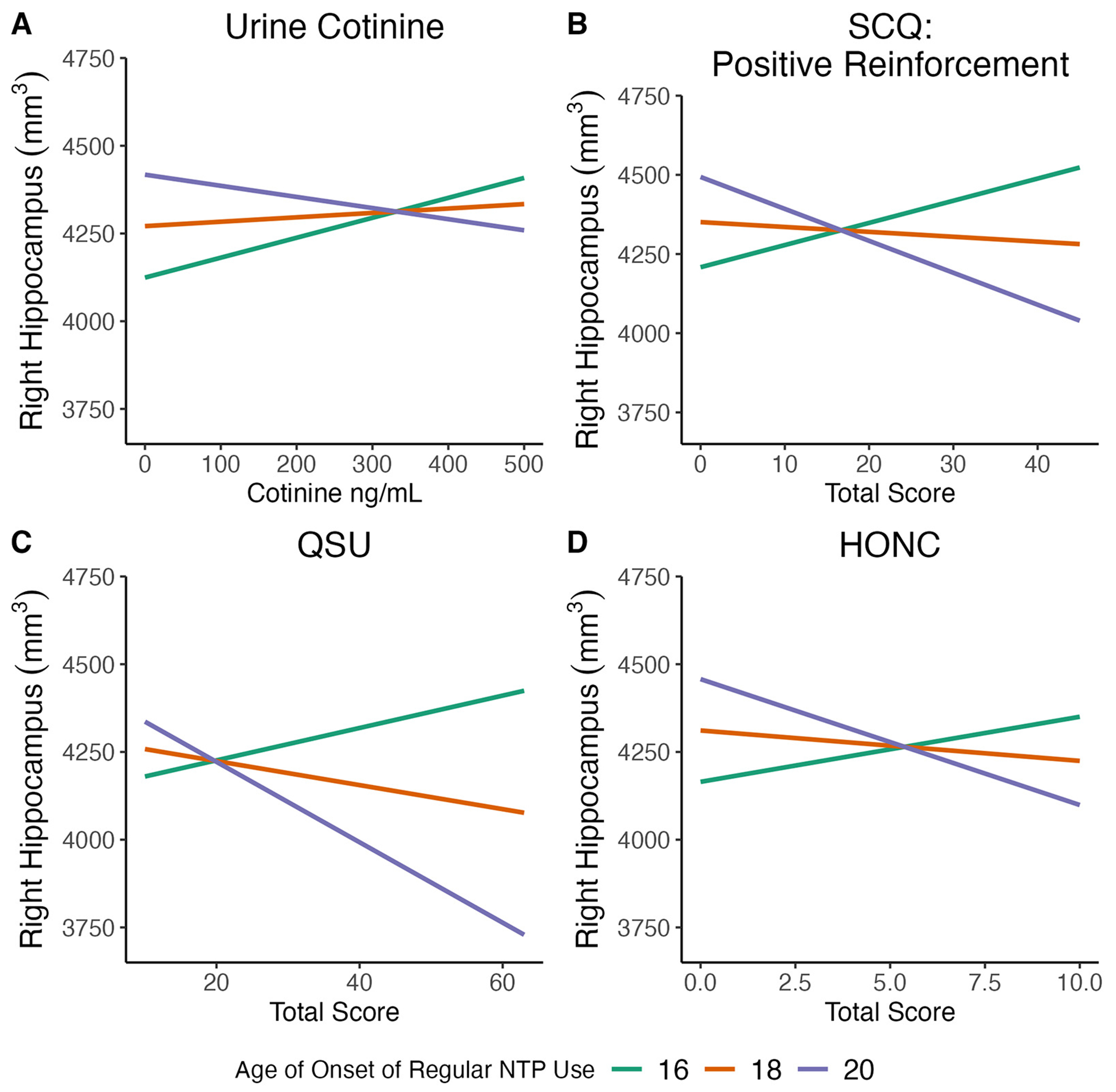
Significant relationships were observed between age of onset of regular use of nicotine and tobacco-related products (NTPs) and measures of nicotine addiction severity on (right) hippocampal volume. Measures of nicotine addiction severity included **(A)** urine cotinine; **(B)** Smoking Consequences Questionnaire (SCQ): positive reinforcement; **(C)** Questionnaire on Smoking Urges (QSU); and **(D)** Hooked on Nicotine Checklist (HONC). Data presented are for visualization purposes only and represent trend lines for age of onset of regular NTP use mean age (18 years old) ± 1 SD (20 and 16 years old, respectively).

Regression models were used to examine the relationship between urine cotinine and age of onset of regular NTP use with bilateral hippocampal volumes, controlling for current age, sex, lifetime alcohol, cannabis, and NTP use, and estimated brain volume. Results indicated a significant age of regular use x cotinine interaction for both the left and right hippocampal volumes (Left: *B* = −0.17, *t* = −2.4, *p* = 0.021; Right: *B* = −0.22, *t* = −2.7, *p* = 0.010) (Figure 1A, left not shown). This inverse relationship suggests that as age of regular use of NTPs became younger, hippocampal volumes were larger as a function of increasing cotinine values. Current age, sex, and lifetime alcohol, cannabis, and NTP use were not significant covariates (*p*s > 0.1), though estimated brain volume was a significant covariate for both left and right volumes (*p*s < 0.0001).

### Smoking consequences questionnaire: positive reinforcement

Regression models were used to examine the relationship between the self-reported positive reinforcing effects of nicotine as measured by the SCQ and age of onset of regular NTP use with bilateral hippocampal volumes, controlling for current age, sex, lifetime alcohol, cannabis, and NTP use, and estimated brain volume. Results indicated a significant age of regular use x positive reinforcement interaction for both the left and right hippocampal volumes (Left: *B* = −2.4, *t* = −2.2, *p* = 0.03; Right: *B*: −4.3, *t* = −3.4, *p* = 0.001) (Figure 1B, left not shown). The inverse relationship indicates that hippocampal volumes increased as a function of younger regular use of NTPs and higher positive reinforcement from NTP use. Current age, sex, and lifetime alcohol, cannabis, and NTP use were not significant covariates (*p*s > 0.2), though estimated brain volume was a significant covariate for both volumes (*p*s < 0.0001).

### Smoking consequences questionnaire: negative reinforcement

Regression models were used to examine the relationship between the self-reported negative reinforcing effects of nicotine as measure by the SCQ and age of onset of regular NTP use with bilateral hippocampal volumes, controlling for current age, sex, lifetime alcohol, cannabis, and NTP use, and estimated brain volume. A trend was observed between age of first regular use and negative reinforcement for the right hippocampal volume (*B* = −2.3, *t* = −1.9, *p* = 0.06), though not for the left (*p* > 0.6). The inverse relationship, though not significant, indicates that hippocampal volumes increased as a function younger age of onset of regular use and higher negative reinforcement from NTP use. Current age, sex, and lifetime alcohol, cannabis, and NTP use were not significant covariates ( *p*s > 0.4), though estimated brain volume was significant (*p* < 0.0001).

### Questionnaire on smoking urges

Regression models were used to examine the relationship between self-reported smoking/vaping urge symptoms as measured by the QSU and age of onset of regular NTP use with bilateral hippocampal volumes, controlling for current age, sex, lifetime alcohol, cannabis, and NTP use, and estimated brain volume. Results indicated a significant interaction between age of regular use and smoking/vaping urges for the right hippocampal volume (*B* = −4.0, *t* = −2.1, *p* = 0.039) (Figure 1C), though no relationship was observed for the left (*p* > 0.3). The inverse relationship observed for the right hippocampus suggests that as age of regular use of NTPs became younger and smoking/vaping urge symptoms increased hippocampal volumes were larger. For the right hippocampus, current age, sex, and lifetime alcohol, cannabis, and NTP use were not significant covariates (*p*s > 0.4), though estimated brain volume was significant (*p* < 0.0001).

### HONC dependence

Regression models were used to examine the relationship between nicotine dependence as measure by the HONC and age of onset of regular NTP use with bilateral hippocampal volumes, controlling for current age, sex, lifetime alcohol, cannabis, and NTP use, and estimated brain volume. Results indicated a significant age of regular use x HONC interaction for the right hippocampal volume (*B* = −0.02, *t* = −3.1, *p* = 0.003) (Figure 1D), though no relationship was observed for the left (*p* > 0.9). The inverse association for the right hippocampus indicates that as age of regular use of NTPs became younger and individuals currently exhibited symptoms of nicotine addiction, hippocampal volumes were larger. For the right hippocampus, current age, sex, and lifetime alcohol, cannabis, and NTP use were not significant covariates (*p*s > 0.1), though estimated brain volume was a significant covariate (*p* < 0.0001).

## Discussion

We previously reported greater 6-month nicotine use was associated with larger bilateral hippocampal volumes in a sample of late adolescents and emerging adults (49). In this follow-up report, we sought to examine whether indicators of more problematic nicotine use, including greater subjective nicotine craving, stronger positive and negative reinforcement, elevated cotinine levels, and earlier age of onset of regular nicotine use would be associated with larger hippocampal volumes. Consistent with our hypotheses, the results revealed a general pattern and interaction such that as age of onset of regular use became younger and symptoms of nicotine addiction became more severe, hippocampal volumes increased. Notably, negative reinforcement, or the alleviation of unpleasant states, was not associated with hippocampal volume in this study, which is consistent with adolescents and emerging adults being less sensitive to the negative effects of nicotine but more sensitive to the rewarding aspects (41).

The hippocampus is implicated in the development and maintenance of substance use disorders (64, 65) by its involvement in modulating reinforcement learning and episodic memory of rewards (52). While few studies have examined the relationship between hippocampal volumes and indices of problematic nicotine use, larger bilateral hippocampal volumes have been associated with worse smoking cessation outcomes in a group of adult cigarette smokers (66). Functional MRI studies similarly suggest enhanced activation of the hippocampus in response to contextual smoking cues (67), while increased resting state functional connectivity between the hippocampus and striatum predicted greater substance use at follow-up in adolescents (68). Like the hippocampus, the dorsal striatum is heavily involved in habit formation (69, 70) and contributes to the development of substance dependence (65, 70). Differences in dorsal striatal regions have been observed to be associated with nicotine dependence symptoms such as craving. In a small sample of emerging adults, larger dorsal striatal volume and surface area was related to higher subjective cigarette craving and craving induced by exposure to smoking cues (71). Similarly, larger putamen volumes were associated with greater lifetime history of cigarette smoking as well as younger age of smoking initiation (72). In this context, the larger hippocampal volumes in the present study being associated with more severe symptoms of nicotine dependence, including craving, could reflect enhanced substance-related reinforcement learning, particularly in those who initiate regular use at younger ages. Overall, these processes may be heightened in adolescents and emerging adults (41) and represent a risk factor for the development of nicotine dependence.

Given the cross-sectional nature of the current study, the causal relationship between hippocampal volume and indices of nicotine dependence cannot be determined. Indeed, the larger hippocampal volumes reported here could be a pre-existing risk factor for initiating nicotine use and subsequent development of nicotine-related problems. However, despite the prevalance of NTP use among AEAs (1, 3, 4) and its addictive nature (19–21), few longitudinal studies have focused on identifying brain morphometry that can predict future use (73). One study reported smaller ventromedial prefrontal cortex gray matter volumes among adolescents predicting smoking initiation and maintenance of smoking behavior at follow-up five years later (74). Smaller amygdala volumes similarly predicted daily smoking as well as being associated with externalizing behaviors (75). Notably, these studies specifically examined traditional cigarette smoking initiation while participants in the present study were primarily e-cigarette users. Moreover, a majority of the present sample also used cannabis, at least minimally. Longitudinal studies suggest larger orbitofrontal cortex volumes may predict adolescents who initiate cannabis use as well as greater sensitivity to rewards at baseline (76). Likewise, adolescents who went on to initiate both heavier cannabis and alcohol use were noted to have increased thickness of the parahippocampal gyrus (77). Thus, while lifetime cannabis use was not a significant factor in the present study, our findings may not align with the few existing studies that focused on individuals who engaged primarily in smoking traditional cigarettes.

The results and conclusions of this study must be considered within its limitations. As noted, this study was cross-sectional in design which limits the ability to make causal interpretations. Longitudinal studies like the Adolescent Brain Cognitive Development (ABCD) Study (78) that have followed adolescents prior to and after initiation of nicotine use are needed to understand the relationships between symptoms of nicotine dependency, age of onset of use, and brain morphometry. Additionally, while participants were recruited for low levels of alcohol use and lifetime alcohol use episodes was a non-significant covariate, the total quantity of alcohol use could possibly have an impact on hippocampal volumes (77, 80, 81). Similarly, the sample size reported here is relatively small and, therefore, the results should be replicated in a larger sample size. Moreover, statistical analyses were not controlled for multiple comparisons, highlighting the somewhat preliminary and exploratory nature of these findings. However, initial findings from ABCD-derived data do suggest that larger hippocampal and parahippocampal morphometry may predict substance use initiation more generally (79).

Overall, the present study revealed a relationship between severity of nicotine dependence symptoms, age of onset of regular use, and hippocampal volumes in a sample of late adolescents and emerging adults. The overall pattern of results indicated that greater nicotine dependence symptom severity and younger age of onset is associated with larger hippocampal volumes. While these findings could be related to enhanced reinforcement and learning of NTP-related habits, they could also reflect a predisposing vulnerability. Greater understanding of these interactions between symptoms of nicotine dependence and age of onset of use as well as their relationship with brain health are essential for informing prevention, intervention, and public health policy.

## Figures and Tables

**TABLE 1:** Sample demographics and characteristics.

Characteristic	*N* = 86^a^
Age	19.9 [16.0–22.0]
% Male	63% (54)
**Race/Ethnicity**	
% White	57% (49)
% Hispanic	34% (29)
Education (years completed)	13.5 [10.0–16.0]
NIH toolbox crystalized composite (age-corrected)	106 [83–146]
Estimated lifetime alcohol episodes	209 [5–978]
Estimated lifetime cannabis use episodes	1,149 [0–14,566]
Days since last cannabis use	40 [0–1,070]
Estimated lifetime NTP use episodes	7,738 [14–87,010]
Age of onset of regular NTP use	18.09 [13.00–22.00] IQR [17, 19]
Years of regular NTP use	1.85 [0.00–7.00]
Days since last NTP use	10 [0–284]
Number of cigarettes previous 6 months	57 [1–1,000]
Urine cotinine (ng/ml)	260 [0–500]
HONC total score	5.1 [0.0–10.0]
SCQ: positive reinforcement total score	13 [0–45]
SCQ: negative reinforcement total score	20 [0–63]
QSU total score	18 [10–63]

a Mean [Range]; % (*n*);

IQR, interquartile range; NTP, nicotine and tobacco-related product; HONC, hooked on nicotine checklist; SCQ, smoking consequences questionnaire; QSU, questionnaire of smoking urges.

## Data Availability

The raw data supporting the conclusions of this article will be made available by the authors, without undue reservation.
